# Posterior Mandibular Displacement—A Systematic Review Based on Animal Studies

**DOI:** 10.3390/ani11030823

**Published:** 2021-03-15

**Authors:** Ioannis Lyros, Miltiadis A. Makrygiannakis, Theodoros Lykogeorgos, Efstratios Ferdianakis, Apostolos I. Tsolakis

**Affiliations:** 1 Department of Orthodontics, School of Dentistry, National and Kapodistrian University of Athens, 11527 Athens, Greece; mimak90@hotmail.com (M.A.M.); stratis-fer@hotmail.com (E.F.); apostso@otenet.gr (A.I.T.); 2 Paediatric Dentist, “Hatzikosta” General Hospital of Messolonghi, 30200 Messolonghi, Greece; theolyk@gmail.com; 3 Department of Orthodontics, Case Western Reserve University, Cleveland, OH 44106, USA

**Keywords:** mandibular growth, mandibular posterior displacement, mandibular length, ramus height, rat

## Abstract

**Simple Summary:**

Extreme growth of the lower jaw is an essential determinant of facial appearance and treatment is challenging. The mandibular joint is important for mandibular growth and backward traction may be applied to address its protrusion. Nevertheless, the conclusions following animal experiments have been contradictory; additionally, joint disorders could arise because of abnormal, traumatic pressure. Our aim was to review the impact on the condyle and the jaw of their distal displacement as found in published research involving rats and rabbits, up to October 2020. In those animals, the mandibular/condylar retraction led to occlusal improvement, but some relapse might be anticipated. The mandibular condyle remained more posteriorly, restriction of further growth was observed, the posterior surface became more flattened, but it became thicker in its neck. The dimensional alterations persisted for the entire period of study and the mandible resumed its inherited growth pattern after the discontinuation of the orthopedic force. Posterior mandibular displacement may be anticipated to produce clinically significant restriction in condylar growth, mainly attributed to remodeling. The properties of the applied force may affect the timing of mandibular formation or just prove traumatic. Outcome stability is a matter of concern and more studies are required to resolve the issue.

**Abstract:**

Treating extreme mandibular growth is challenging. The mandible is pushed backwards to address itsprotrusion. Nevertheless, conclusions after such displacement in animals have been contradictory. The aim of the present review is to present measurable alterations of the mandible and the condyle following retractionin healthy rats or rabbits. PubMed, Scopus and Web of Science were accessed for relevant studies up to October 2020. Eligibility was determined by the PICOS process, while the risk of bias was estimated with SYRCLE’s risk of bias tool. Retraction resulted in a more distal molar occlusion and the condyle rested more posteriorly. Mandibular anteroposterior bilateral growth restriction was achieved, the condylar process measured smaller and its angulation increased. The condylar neck thickened, its posterior surface flattened, the coronoid process was measured longer, and enlarged retromolar density was registered. Differences in the ramus height and the intercondylar distance were insignificant. Changes persisted for the period of study and subsequently the mandible resumed its inherited growth pattern. The timing of mandibular shaping and TMJ outcomes might depend on the properties of the applied force. Stability is of concern and well-structured, long-term studies are expected to resolve the issue and further clarify the results of posterior mandibular displacement.

## 1. Introduction

The scope of orthodontics is to elucidate craniofacial growth, treat predictably the skeletal discrepancies and to align the dentition [1]. To achieve these goals, researchers get motivated to understand the development and function of the bony tissue and the temporomandibular joint (TMJ) alike. It is noteworthy that facial appearance may affect self-esteem [2,3] and quality of life [4], hence the orthodontist seeks either to prevent or diagnose early, and then to tackle the most prominent malformations [5].

Treatment in cases of extreme mandibular growth has been a challenge [6,7] and research has focused on the anatomy, histology and function of the TMJ [8,9,10] seeking the trigger of growth [11]. In experiments and in clinical practice, the mandible has been pushed backwards, mainly during the period of growth, for protrusion to alleviate [12,13,14]. Clinical observations of animal TMJs and the consequent suggestions after mandibular displacement have been heterogeneous and contradictory. Others claim potential for TMJ disorders and joint structural alterations due to the generation of parafunctional stress, deemed as traumatic [15,16,17].

Mandibular condyle is covered by cartilage, consisting of cellular components in extracellular matrix composed of fibrous (mainly collageneous) elements and proteoglycan aggregate [18]. The unique structure of the condylar cartilage comprises distinct layers [19], capable of adaptive remolding in response to masticatory function and external loading [20,21,22]. The condylar cartilage is mainly a load-bearing structure for induced biomechanical stress and its thickness has been suspected to undergo functional adaptation [23]. The TMJ performs complex hinge and sliding movement [8]. During mastication, compressive, shearing, and other complex forces are exerted on the mandibular condyle [21].

Condylar growth is affected by heredity [24,25,26,27], hormones [28,29,30,31,32], the environment [33,34], systemic diseases [35,36,37] and stress [38,39] and is significant in the development of the orofacial complex [40]. Customary mastication consists a physiological stress to the TMJ, of great importance for its development in adolescence and the remodeling in adulthood [41]. The lateral condylar displacement in the glenoid fossa as observed in the therapeutic approach of skeletal discrepancies may culminate in abnormal loading of adjacent structures, affecting the physiologic dynamics of condylar cartilage and triggering the release of growth factors [19,42] and inflammatory mediators [43], to unknown extent and of unspecified clinical significance, a long-standing controversy. 

Articular dysfunction may have adverse consequences on the potential for remodeling, resulting in histological alterations and changes in condylar volume. As a result, mandibular retrusion may lead to adverse outcomes in cartilage formation, as has been reported in rats, suggesting dysfunction and disarrangement [16,43,44]. However, others claim that TMJ disorder should not be an issue [45]. Clinical investigations of the effect of orthodontic mandibular displacement in humans during treatment of malocclusion have suggested that the results of treatment appear to be achieved mainly by remodeling of the TMJ [17,46,47].

The present study aims to review the impact of distal mandibular dislocation on the bony and cartilaginous component of the condyle. Currently, the rat is preferred as experimental model, although earlier studies have studied monkeys as well [48,49]. Additionally, the rabbit [44], the dog and artiodactyl mammals have been proposed as suitable models for studying TMJ dysfunction [50]. The present review comprises studies on rodents (including rabbits) despite existing anatomical and functional differences with humans [51,52]. The present investigation presents potential structural condylar changes due to mandibular distal displacement and aspires to gain further insight on the effect of increased mechanical stimulation on cellular response and growth within the condylar structures. 

This review aimed to systematically appraise the quality of the available evidence in animal studies regarding the effects (macroscopic, measurable, dimensional changes) following posterior displacement of the mandible.

## 2. Materials and Methods

A specific protocol was developed and piloted according to the guidelines in the PRISMA-P statement [53]. The Cochrane Handbook for Systematic Reviews of Interventions [54] and the PRISMA statement [55] were followed.

### 2.1. Eligibility Criteria

Eligibility criteria were formulated according to the PICOS (Participants, Intervention, Comparison, Outcomes and Study design) process (Supplementary Table S1). Relevant research involved healthy animals sustaining backward mandibular displacement. Review and meta-analytic articles were not regarded eligible.

### 2.2. Information Sources and Search Strategy

Three databases (PubMed, Scopus, Web of Science) were used to identify all relevant studies independently of language, date or status of publication. They were searched since inception up to October 2020. Two authors (I.L. and M.A.M.) produced comprehensive search procedures, appropriately modified to tackle nuances in vocabulary and syntax (Supplementary Table S2). 

In addition, reference lists were searched meticulously for further studies to surface. The authors were to be contacted for additional data.

### 2.3. Study Selection

The first (I.L.) and third (T.L.) authors assessed the retrieved records independently and in duplicate. Although they were not blinded to the identity of the authors or the conclusions of the studies, they used the same method to assess the eligibility of all retrieved records. All doubts were resolved by discussion with the fifth co-author (A.I.T.).

### 2.4. Data Collection

Authors I.L. and T.L. conducted data extraction. A customized data collection form was created and used to gather the following information from the selected studies: study details, each design and eligibility verification, features of the subjects and the appliances used, the intervention itself, duration of treatment and outcomes.

### 2.5. Risk of Bias in Individual Studies

It was assessed by I.L. and M.A.M. in duplicate with the SYRCLE’s risk of bias tool (2014) [56], and according to Higgins and Green [54]. Arising disputes were discussed with A.I.T.

### 2.6. Summary Measures and Shaping of Results

Quantitative data synthesis for meta-analysis was not performed as initially envisioned, due to inadequate data on outcomes and the differences in the respective methods [54,57,58].

## 3. Results

### 3.1. Study Selection

**Figure 1:**
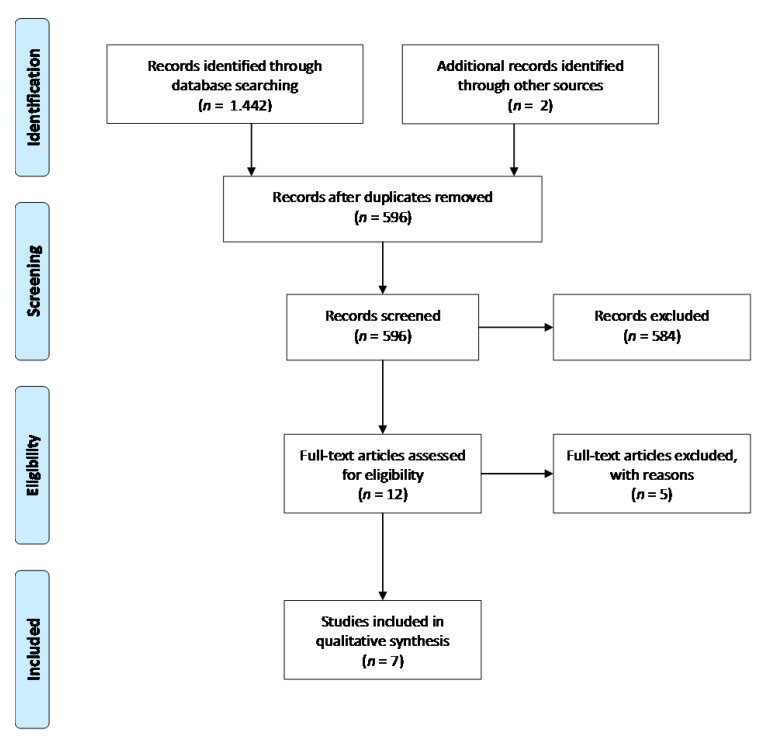
Flow of records through the reviewing process.

Figure 1 summarizes the cascade of the reviewing process. A total of 1444 records were shortlisted from the initial search (2 originating from reference lists). From them, 848 were excluded as duplicates and further 584 following assessment of title and abstract. From the remaining 12 studies, 5 were excluded, due to absence of radiologic macroscopic data following posterior displacement of the mandible. Finally, 7 full-text records comprised the systematic review [12,16,18,42,44,59,60].

### 3.2. Study Characteristics

Table 1 presents the characteristics of the included studies. The length of the period of study ranged from 1 day to 16 weeks. The majority of the included studies (5) had used male Wistar growing rats as experimental animals, but there was also one study that had reported on female Wistar rats and another that had recruited rabbits.

Orthodontic/orthopedic treatment was induced by adapting intraoral or/and extraoral appliances. Orthopedic collar appliances were used by Asano [59], Desai et al. [44] cemented castings on maxillary incisors, Teramoto et al. [18] manufactured a collar from acrylic plate and rubber band connected to the mandibular incisors with wire jig and coil, and occlusal guiding appliances were attached to the maxillary incisors by Cholasueksa et al. [16] and by Farias-Neto et al. [12], while both Hua et al. [60] and Wang et al. [42] introduced a twin inclined plane device. 

Treatment was evaluated mainly by linear measurements of the mandible. Some of them studied molar relationship [16,44] and one of them, the position of the mandibular condyle [18].

### 3.3. Risk of Bias within Studies

The outcomes of the risk of bias assessment are summarized in Table 2. Four studies were deemed as being of high [18,42,44,59], and three of unclear risk of bias [12,16,60]. Regarding allocation sequence, four of them were found to be of high risk of bias, while unclear risk of bias was detected for the rest of them. Most of the studies were of unclear risk of bias considering allocation concealment, caregivers’ blinding and assessors’ blinding. Only three of them used comparable animal clusters regarding gender, age, and weight and thus were assessed of low risk of bias. Data concerning randomization of animal housing remained unclear. The risk of bias related to the animal random selection for the outcome assessment was considered to be unclear for all of them as well. In terms of handling of incomplete data and selective outcome reporting, the risk of bias was rated as unclear for all of them. Overall, details in the studies were insufficient to uncover further issues linked to the risk of bias.

### 3.4. Results of Individual Studies

In growing Wistar rats, the mandible grew shorter anteroposteriorly, the coronoid process became higher, the condylar neck measured thicker and an enlarged retromolar corpus was evident after application of anorthopedic collar device exerting a backward force on the mandible [59]. Moreover, the orthopedic effects were limited to the period when the force was applied and the mandible returned to the inherited growth pattern in both the experimental and control groups after the activation of the appliance had ceased, when the mandibles resumed growing at similar rates, in anteroposterior direction, as regulated by genetics [59]. Indeed, the mandibular area where growth was more pronounced due to the intervention showed less subsequent growth and remodeling comparing with controls [59]. Allegedly, the above-mentioned alterations in mandibular development had not affected the overall growth pattern as evaluated by skull dimensions and body weight [59]. Notwithstanding, mandibular retraction did not significantly affect the condylar height and the thickness of the angular process [59]. In another similar experiment involving rats, it was confirmed radiographically that the posterior mandibular displacement prevented the mandibular condyle from displacing anteriorly in the temporal fossa [18].

In rats and rabbits subjected to mandibular/condylar backward retraction with the aid of inclined planes cemented on maxillary incisors or properly modified guiding appliances, lateral radiography disclosed mandibular molars occluding more distally in relation to the maxillary [16,44]. Interestingly however, at a later age the radiographic, distalized molar relationship became less pronounced, supposedly an attempt on the part of the subject to establish a new balance within the altered oral environment [44].

Mandibular posterior displacement in growing rats by an appropriate occlusal guiding appliance attached to the maxillary incisors resulted in shorter mandibular length on both sides [12]. In the experimental group, statistically significantly smaller mandibular lengths were measured radiographically, without any noteworthy difference between the left and right sides [12]. Additionally, statistically insignificant differences were observed between experimental groups regarding the ramus height and the intercondylar distance [12].

The cementation of modified inclined planes on the upper and lower molars of rats effected a statistically significantly shorter condylar process and significantly larger angulation of its axis to the mandibular plane in the experimental groups, as evidenced radiographically [60]. Both the overall mandibular length and the condylar height remained significantly smaller in the experimental groups compared to the controls until the end of the period of study [60]. By the end of the experiment, also the condylar width measured significantly less in test subjects [60]. By the midst and the end of the observation period, the condylar posterior surface appeared flattened compared to that of the control groups; additionally, its most posterior point had shifted upward [60]. Eventually, the decrease in mandibular length in experimental animals was attributed to the remodeling of the condyle [60]. Ultimately, Wang et al. [42] found in the rat that a twin inclined device resulting in posterior mandibular displacement may lead to adaptive bone resorption at the posterior region of the condyle. In their control group, the posterior margins of the condylar bone remained round, whereas in the experimental group the lower part of the posterior margin of the condyle appeared significantly flattened by the end of the observation period as highlighted by 3D reconstruction [42].

## 4. Discussion

### 4.1. Summary of Evidence

Orthodontics aims to study and guide the growth and development of maxillofacial elements and proportions that contribute to normal appearance and functional demands [5]. These are essential for basic functions, namely mastication, swallowing and breathing [61,62,63]. Thus, the ongoing interest in the growth of the maxilla and the mandible may not come as a surprise [64]. Even conservative, non-surgical interventions may affect the functioning of the TMJ and facial appearance. 

Knowledge on mandibular growth is acquired by longitudinal clinical studies in normal individuals as well as by experiments that use various animal models, mainly primates [48,49], rodents and other mammals [51]. The rationale for selecting the rodents has been a matter of debate. It is speculated that the existing anatomical differences with humans may lead to erroneous conclusions. On the other hand, higher financial costs seem to limit experimentation with non-human primates; additionally, current restrictions imposed by ethics prevent recruiting humans as experimental subjects in interventions that may culminate in irreversible or undesirable outcomes [65]. There has not been a definitive agreement on the mechanism [14] and the possible side effects of the most common treatments of developmental deviations [43,45,66]. 

In the present systematic review, we delved into published experimental studies involving rats and rabbits that report on the effects of such a common conservative approach as in cases of extreme mandibular growth, namely the application of retrusive force on the mandible. The focus was set on research with radiographic, macroscopic outcomes and thus, seven papers were short-listed. Interestingly, most of them included mainly histological and biochemical observations that potentially accompany the mandibular retrusion [16,18,42,44,60]. Although radiography is preferred in daily dental practice as crucial diagnostic tool either for orthodontic reasons [67,68] or complex therapeutic schemes like implant placement and sinus floor elevation [69], in some of the selected studies, lateral radiography seems to have been used only to confirm the backward displacement of the mandible in experiments focusing mainly on cellular and molecular changes [16,18,44].

The predominant animal in the studies of our review was the rat [12,16,18,42,59,60], although Desai et al. [44] experimented on rabbits and earlier studies have selected the monkey. The age of the animals is of importance because mandibular growth is related to general growth, and varies in relation to chronological age [6,70,71]. The age of the animals was clearly reported, but varied from 4 weeks [59], 5 weeks [12], 6 weeks in the papers by Hua et al. [60] and Wang et al. [42] up to 8 weeks [16,18], and even 9 months in rabbits [44]. Moreover, bone turnover depends on sexual hormones; additionally, TMJ pathology has been linked to the hormonal profile [31]. In the present review, five studies reported on male Wistar rats [16,18,42,59,60], while Farias-Neto et al. [12] experimented on female Wistar rats and Desai et al. [44] on rabbits of unidentified sex. The method to produce the mandibular displacement included inclined planes cemented on maxillary incisors [12,16,44] or the molars [42,60], and collar extraoral appliances exercising orthopedic traction by attachments on the lower incisors [18,59]. All the included studies had control and experimental groups and the comparison was performed between them to identify differences of statistical significance at the level of 5%, at least. Statistical methodology was stated without much detail, particularly addressing the aspect of statistical normality. Inadequate or inappropriate statistics may contribute to systematic errors and thus potentially undermine the quality of conclusions of the present systematic review.

Dental professionals treating patients would like to know whether a given treatment modality involving posterior mandibular dislocation has a stable effect on the net growth and consequently the facial dimensions, which affect the appearance [6]. Nevertheless, the studies being reviewed here are heterogeneous regarding their outcomes and they predominantly report on histology and biochemistry despite the use of lateral radiography. 

Unfortunately, lateral radiography was rather used to confirm the change in the relationship between the maxillary and mandibular molars in the studies by Cholasueksa et al. [16] and by Desai et al. [44]. Indeed, in both, the mandibular first molars in the experimental group moved in a distal position relative to the maxillary ones after posterior displacement of the mandible. In addition, the customary posterior mandibular displacement was considered dysfunctional and traumatic as evidenced by the production of proteins indicating damaged nerve fibers in the retrocondylar region [16]. Similarly, Desai et al. [44] found alterations, albeit statistically insignificant, in the spatial orientation of the temporomandibular disk that allegedly might predispose to anterior disk displacement concomitant with TMJ disorder [44]. Anterior displacement of the articular disk was proposed by Teramoto et al. [18], who also found that the condyle in their experimental group sustaining backward compressive force was positioned more posteriorly within the articular fossa during mouth opening, compared to the control group.

Cephalometric measurements by Asano [59] showed that the mandibles that are pushed backward end-up smaller in length, having less volume and weight. He calculated an increase in the size of the anterior mandibular region, coronoid process, the neck of the condyle and also found thickening of the retromolar region in the experimental group in relation to controls. However, the condylar height and the thickness of the angular process remained statistically unaffected. The observed differences occur due to localized differential bone apposition and resorption leading to remodeling and adaptation to accommodate the applied force in the altered environment. 

An important finding was that the differences in growth remained after the cessation of the external force and growth direction returned to the inherited growth behaviour, meaning that a lasting effect may be anticipated in similar cases [59]. It is noteworthy that the use of various experimental devices was not found to have any significant influence to general growth or the size of the skull as a whole [12,59,60]. In agreement with the aforementioned research, Farias-Neto et al. [12] also found decreased mandibular length in cases of functional posterior mandibular displacement, but negligible difference in the height of the ramus. Farronato et al. speculated that differences in condylar heads could be attributed to condylar growth center dysregulation, whereas the reduced condylar neck volume could have been an outcome of growth deficit and also the height of the ramus had not been significantly affected [72].

Similarly, Hua et al. [60] reported that when inducing backward movement of the mandible, reductions in the length of the condylar process and the mandible may be expected. Their cephalometric analysis revealed a greater increase of the angle of the condylar process to the mandibular plane and a decrease of the condylar width in the experimental animals. Moreover, they mentioned that the most posterior condylar point had shifted upward and the posterior condylar surface had a tendency to flatten, indicating bone resorption [60]. Flattening of the entire posterior margin of the condyle became progressively evident and statistically significant compared to controls in the study by Wang et al. [42], who experimented in the rat with posterior inclined planes that apply a functional retrusive force. This pattern of change is compatible with progressive adaptation of the condylar bone to mild, continuous and progressive pressure [42].

The observed changes of the various mandibular regions may be attributed to the remodeling that happens due to the paranormal, dysfunctional external force and the potential consequent loss of the optimal, customary mastication force by restricted mandibular movement. The explanation of the mechanism that leads to such an outcome should be sought out within molecular pathways and in cellular interactions [12,18]. In humans, the differences may be expected more pronounced than in the rodents; additionally, bone resorption may be anticipated in the posterior condylar surface and the anterior region of the post glenoid eminence, because of existing anatomical differences [12,16,60]. Nevertheless, in growing individuals the ultrastructural changes in the posterior area of the condyle due to mechanical stress could be anticipated to reverse spontaneously at earlier stages [42]. It is of interest that children in whom breastfeeding had persisted for more than 6 months could be considered as less likely to develop malocclusion in primary dentition [73].

Lastly, our literature search disclosed one more piece of research for which only the abstract could be retrieved despite all efforts and thus was not included in the present review. It mentions, in agreement with the above conclusions, that the mandibular backward movement effected by an inclined-plane appliance, produces a significant increase of the angle between the condylar process and the mandibular plane, and a noteworthy decrease in condylar width. Additionally, the posterior condylar surface was found flattened in the experimental group, while the length of the mandibular base and the distance of the condylar head to the mandibular plane did not alter significantly. Therefore, it was concluded that the above functional intervention might inhibit the growth of the condyle and the mandible [74].

### 4.2. Strengths and Limitations

The present systematic review was based on well-established guidelines as outlined in the Materials section. The searching procedure was extensive, including digital and printed literature, up to October 2020, and was detailed including every potentially eligible report, even assessing the lists of references of relevant articles. Every possible attempt was made to diminish bias, by screening, verification of eligibility, abstraction of information, as well as assessment of risk of bias and quality of evidence. Dissimilar views were discussed among authors. 

Limitations of the present review might be associated with the nature of the included research and the data characteristics, which culminated in a rather low level of evidence. The shortage of relevant data and the fact that the outcomes were predominantly histological and biochemical, not measurable but descriptive, prevented the conduct of further meta-analysis. 

The included reports were assessed as being of unclear or high risk of bias due to inadequate methodology. Uncertainty pertaining statistical testing and lack of power calculations was increased by findings that the respective authors had not related their interpretation to clinical practice or had based them on speculations. Above all, it should be highlighted that the conclusions reached in the included studies have been based on animal research and thus may not be fully applicable to humans due to differences in anatomy and physiology. The observed methodological heterogeneity further precludes adopting spontaneously the retrieved information for human clinical scenarios.

### 4.3. Recommendations for Future Research

Because individuals featuring visible developmental aberrations pertaining to mandibular prognathism may have apparent need for treatment, it is of importance to ascertain the mechanism of action and to clarify the potential side effects of the intervention studied in the present systematic review. Based on the observations in animal studies and those in the present review, the orthodontic community may call for funding to organize high quality controlled studiesin accordance with ethical guidelines in order to provide evidence based, definite, measurable and simple to interpret conclusions on the issue of controlling mandibular growth, with emphasis to long-term stability.

## 5. Conclusions

Considering the aforementioned evidence and its limitations, we dare to conclude that the procedures investigated in the animal experimental studies seem to have clinically significant restrictive anteroposterior growth effects in the mandible. The available evidence shows that various appliances exert distal mandibular pressure or maintain functional, intermittent, backward intraoral guidance. It seems that the resulting outcomes of mandibular distal displacement are rather stable overtime, although to unspecified extent. More high-quality studies are necessary in order to further clarify the effect of posterior displacement of the mandible.

## Figures and Tables

**Table 1:** Features of the included reports.

Articles	Population	Intervention	Compared with	Outcome of Interest of Studies	Method of Assessment	Results
Asano, 1986	180 M, 4w-old Wistar rats	Orthopedic collar appliances for mandibular retractive force (8 h/d)	20 rats in each group.	(1) 3D alterations on the growing mandible after retractive mandibular force		
			EG1:collar appliance with retractive force for 8w,		Radiographic data	Ø Volume and length of the mandibles: EG1 < CG1.
			EG2:10w, EG3:12w, EG4:16w	(2) mandibular growth after the orthopedic force was removed		Ø Height of anterior region and coronoid process, thickness of the retromolar corpus and condylar neck: EG1 > CG1.
						Ø Skull, condylar height and thickness of angular process: EG ≈ CG.
			CG0:collar appliance without retractive force for 4w,			Ø Bone deposition lingually and buccally during force application: EG ≈ CG.
			CG1:8w, CG2:10w, CG3:12w, CG4:16w			Ø Bone deposition on the lingual surface EG > CG
						Ø Bone deposition on the buccal surface EG < CG.
Cholasueksa et al., 2004	39 M, 8w-old Wistar rats	Intermittent, functional posterior condylar displacement with modified guiding appliance attached to maxillary incisors	EG:24 rats, CG:15 rats, EG1:appliance for 4d,	Remodeling process of the TMJ	Lateral radiographs	
			EG2:7d,			Ø Distal relationship of mandibular first molars compared to maxillary: EG > CG
			EG3:14d			Ø EG1,2,3: no incisal attrition of the mandibular incisors
	
			CG1:4d without appliance, CG2:7d,			
			CG3:14d			
Desai et al., 1996	8, 9m old New Zealand white rabbits	Inclined planes on maxillary incisors. Functional continuous posterior mandibular displacement for 33 d	EG1:appliance for 2d,	TMJ morphological and spatial changes	Incisal relationships Radiographic data (lateral head X-rays)	Distalization of mandibular molars:
			EG2:7d,			Ø EG1 > CG1
			EG3:33d			Ø EG3 < EG1
	
			CG1:2d,			
			CG2:7d,			
			CG3: 33d			
Farias-Neto et al., 2012	20 F, 5w-old Wistar rats	Functional mandibular posterior displacement with occlusal guiding appliance attached to maxillary incisors	EG1:10 rats, appliance for 8w (Right side studied), EG2:the same 10 rats of EG1, appliance for 8w, (Left side studied)	Mandibular growth	Scan images with classic i-CAT and acrylic rapid prototyped templates of the mandibles	Ø Mandibular length: EG1,2 < CG,
			CG: 10 rats without appliance for 8w, sham operation			EG1 ≈ EG2
						Ø Ramus height and intercondylar distance between groups and sides: EG1,2 ≈ CG
						Ø Altered mandibular bone morphology at grown age
Hua et al., 2012	8 M, 6w-old Wistar rats	Gradually induced backward movement of the mandible by a twin inclined plane device bonded to the posterior teeth	EG1:8 rats, device for 3d, EG2:8 rats, 14d, EG3:8 rats, 30d, EG4:8rats, 60d	Mandibular condyle remodeling	Radiographs and true-color video camera	Condylar remodeling
			CG1:4 rats, 3d, no device,			Ø Length of condylar process, the dependent mandibular length and the condylar length: EG1,2 ≈ CG1,2; EG3 < CG3; EG4 < CG4
			CG2:4 rats, 14d, CG3: 4 rats, 30d, CG4:4 rats, 60d			Ø Length of mandibular base: EG1,2,3,4 ≈ CG1,2,3,4
						Ø Angle of the condylar process axis to the mandibular plane: EG1,2 ≈ CG1,2; EG3 > CG3; EG4 > CG4
						Ø Condylar width: EG1,2,3 ≈ CG1,2,3; EG4 < CG4
						Ø Flattening of the posterior condylar surface: EG3 > CG3; EG4 > CG4
						Ø Upwards shifting of the most posterior point of the condyle: EG4 > CG4
Teramoto et al., 2003	24 M, 8w-old Wistar rats	Continuous compressive loading of the TMJ	EG1:7 rats appliance for 7d, EG2:5 rats for 1d, EG3:5 rats for 3d	Effects of compressive forces on extracellular matrix of mandibular condylar cartilage	Radiographic analysis (soft X-ray)	Ø EG1,2,3: the condyle remained under the articular eminence
			CG: 7 rats, not treated			Ø CG: mandibular condyle moved anteriorly
Wang et al., 2019	48 M 6w-old Wistar rats	Twin inclined plane device bonded to the posterior teeth to effect posterior mandibular movements	EG1:8 rats, appliance for 3d, EG2:8 rats, 14d, EG3:8 rats, 30d, EG4:8 rats, 60d	Posterior condylar area	Morphometric analysis by microcomputed tomography (micro-CT)	Flattening of the posterior region of the condyle
			CG1: 4 rats, no appliance for 3d CG2: 4 rats, 14d, CG3:4 rats, 30d, CG4:4 rats, 60d			Ø CG1 ≈ CG2 ≈ CG3 ≈ CG4 ≈ EG1 ≈ EG2
						Ø Lower part EG3 > EG1,2
						Ø Superior part: EG3 ≈ EG1,2
						Ø Entire posterior margin: EG4 > EG3

CG: control group, EG: experimental group, F: female, M: male, h: hour, d: day, m: month, w: week, < or >: statistically significant difference, ≈: statistically non-significant difference.

**Table 2:** Summary of risk of bias assessment.

	Signaling Questions										
**Study**	**1**	**2**	**3**	**4**	**5**	**6**	**7**	**8**	**9**	**10**	**Summary**
Asano, 1986	High	Low	Unclear	Unclear	Unclear	Unclear	Unclear	Unclear	Low	Unclear	High
Cholasueksaet al., 2004	Unclear	Low	Unclear	Unclear	Unclear	Unclear	Unclear	Unclear	Low	Unclear	Unclear
Desai et al., 1996	High	Unclear	Unclear	Unclear	Unclear	Unclear	Unclear	Unclear	Low	Unclear	High
Farias-Neto et al., 2012	Unclear	Unclear	Unclear	Unclear	Unclear	Unclear	Unclear	Unclear	Low	Unclear	Unclear
Hua et al., 2012	Unclear	Unclear	Unclear	Unclear	Unclear	Unclear	Unclear	Unclear	Low	Unclear	Unclear
Teramotoet al., 2003	High	Unclear	Unclear	Unclear	Unclear	Unclear	Unclear	Unclear	Low	Unclear	High
Wang et al.,2019	High	Low	Unclear	Unclear	Unclear	Unclear	Unclear	Unclear	Low	Unclear	High

1: Was the allocation sequence adequately generated and applied?; 2: were the groups similar at baseline or were they adjusted for confounders in the analysis?; 3: was the allocation adequately concealed?; 4: were the animals randomly housed during the experiment?; 5: were the caregivers and investigators blinded to the intervention that each animal received?; 6: were animals selected at random for outcome assessment?; 7: was the outcome assessor blinded?; 8: were incomplete outcome data adequately addressed?; 9: are reports of the study free of selective outcome reporting?; 10: was the study apparently free of other problems that could result in high risk of bias?

## Data Availability

The data presented in this study are available in the included studies of this systematic review.

## Supplementary Materials

The following are available online at https://www.mdpi.com/2076-2615/11/3/823/s1, Table S1: Eligibility criteria for the present systematic review; Table S2: Strategy for database search (up to October 2020).
