# Supplementary material for: Posterior Mandibular Displacement—A Systematic Review Based on Animal Studies
Source: Animals (Basel). 2021 Mar 15;11(3):823. doi: 10.3390/ani11030823 (PMC8000001; doi:10.3390/ani11030823)
Supplement: Supplementary file 1 [file animals-11-00823-s001.pdf]

Supplementary Table1. Eligibility criteria for the present systematic review.

Domain	Inclusion criteria	Exclusion criteria
**Participants**	▪ Animal subjects (rodents) undergoing orthodontic/orthopaedic treatment, concerning backward movement of the mandible	▪ Primate animal species (i.e. macaca mulatta) undergoing orthodontic/orthopaedic treatment
**Interventions**	▪ Orthodontic/orthopaedic devices effecting posterior displacement of the mandible	
**Comparisons**	▪ Placebo intervention or no intervention	
**Outcomes**	▪ Quantitative macroscopic data regarding orthodontic/orthopaedic treatment [i.e. mandibular length change] measured mainly by radiography [lateral cephalometric radiographs, Cone Beam CT, micro-CT etc.]	▪ Histological, histochemical, immunohistochemical, qualitative assessments regarding orthodontic/orthopaedic treatment [i.e. histologic data or immunohistochemistry]
**Study design**	▪ Experimental prospective controlled studies (according to the Scottish Intercollegiate Guidelines Network algorithm for classifying study design (available at http://www.sign.ac.uk/ assets/study_design.pdf)	▪ Reviews, systematic reviews and meta-analyses

Supplementary Table2. Strategy for database search (up to October 2020).

**Database**	**Search strategy**	**Hits**
**General Sources**		
**PubMed** http://www.ncbi.nlm.nih.gov/pubmed	(rat OR mouse OR mice OR rabbit) AND ("mandibular posterior displacement" OR "posterior displacement" OR "guiding appliance" OR "backward movement of the mandible" OR "distal movement" OR "distal displacement" OR "inclined plane" OR "twin inclined plane")	**421**
**Scopus** https://www.scopus.com/search/form.url?zone=TopNavBar&origin=searchbasic	TITLE-ABS-KEY: ((rat OR mouse OR mice OR rabbit) AND ("mandibular posterior displacement" OR "posterior displacement" OR "guiding appliance" OR "backward movement of the mandible" OR "distal movement" OR "distal displacement" OR "inclined plane" OR "twin inclined plane"))	**526**
**Web of Science™** http://apps.webofknowledge.com/	TITLE: ((rat OR mouse OR mice OR rabbit) AND ("mandibular posterior displacement" OR "posterior displacement" OR "guiding appliance" OR "backward movement of the mandible" OR "distal movement" OR "distal displacement" OR "inclined plane" OR "twin inclined plane")) Timespan: All years; Search language=Auto	**495**
**Grey literature sources**		
Reference lists	"mandibular posterior displacement"	**2**
